# Interferon lambda 4 (IFNL4) gene polymorphism is associated with spontaneous clearance of HCV in HIV-1 positive patients

**DOI:** 10.1590/1678-4685-GMB-2015-0106

**Published:** 2016-07-25

**Authors:** Camila Fernanda da Silveira Alves, Camila Schultz Grott, Vagner Ricardo Lunge, Jorge Umberto Béria, Daniela Cardoso Tietzmann, Airton Tetelbom Stein, Daniel Simon

**Affiliations:** 1 Programa de Pós-Graduação em Biologia Celular e Molecular Aplicada à Saúde, Universidade Luterana do Brasil (ULBRA), Canoas, RS, Brazil; 2 Programa de Pós-Graduação em Saúde Coletiva, ULBRA, Canoas, RS, Brazil; 3 Curso de Medicina, ULBRA, Canoas, RS, Brazil; 4 Universidade Federal de Ciências da Saúde de Porto Alegre (UFCSPA), Porto Alegre, RS, Brazil

**Keywords:** IFNL4 genotypes, HIV/HCV co-infected patients, Spontaneous clearance

## Abstract

Approximately one-third of the individuals infected with human immunodeficiency virus type 1 (HIV-1) are co-infected with hepatitis C virus (HCV). Co-infected patients have an increased risk for developing end-stage liver diseases. Variants upstream of the IFNL3 gene have been associated with spontaneous and treatment-induced clearance of HCV infection. Recently, a novel polymorphism was discovered, denoted IFNL4 ΔG > TT (rs368234815), which seems to be a better predictor of spontaneous clearance than the IFNL4 rs12979860 polymorphism. We aimed to determine the prevalence of the IFNL4 ΔG > TT variants and to evaluate the association with spontaneous clearance of HCV infection in Brazilian HIV-1 patients. The IFNL4 ΔG > TT genotypes were analyzed by polymerase chain reaction followed by restriction digestion in 138 HIV-1 positive patients who had an anti-HCV positive result. Spontaneous clearance of HCV was observed in 34 individuals (24.6%). IFNL4 genotype distribution was significantly different between individuals who had spontaneous clearance and chronic HCV patients (p=0.002). The probability of spontaneous clearance of HCV infection for patients with the IFNL4 TT/TT genotype was 3.6 times higher than for patients carrying the IFNL4 ΔG allele (OR=3.63, 95% CI:1.51-8.89, p=0.001). The IFNL4 ΔG > TT polymorphism seems to be better than IFNL4 rs12979860 to predict spontaneous clearance of the HCV in Brazilian HIV-1 positive patients.

Human immunodeficiency virus type 1 (HIV-1) infects approximately 38 million people worldwide. About one-third of these individuals are also co-infected with hepatitis C virus (HCV) because of the shared transmission routes (Soriano *et al.*, 2007; World Health Organization, 2013). Co-infected patients have an increased risk for developing end-stage liver diseases, including cirrhosis and hepatocellular carcinoma (Soriano *et al.*, 2013).

Genome-wide association studies (GWAS) identified single nucleotide polymorphisms upstream of interferon-λ3 gene (IFNL3; formerly known as IL28B), strongly associated with spontaneous and treatment-induced clearance of HCV infection (Ge *et al.*, 2009; Suppiah *et al.*, 2009; Tanaka *et al.*, 2009; Rauch *et al.*, 2010). The IFNL3 gene, located on chromosome 19, encodes interferon-λ3 protein, a cytokine which has antiviral properties *in vitro* and *in* vivo (Hou *et al.*, 2009) through activation of the JAK-STAT pathway and up-regulation of interferon-stimulated genes (ISGs) (Zhou *et al.*, 2007). Several studies confirmed the significant association between the CC genotype of the rs12979860 polymorphism (located approximately 3Kb upstream of IFNL3) and spontaneous and treatment-induced clearance of HCV infection in both HCV mono-infected and HIV/HCV co-infected patients (Matsuura *et al.*, 2014). However, it was not clear how the genotypes of the rs12979860 polymorphism affect the interferon signaling pathways (Balagopal *et al.*, 2010; Prokunina-Olsson *et al.*, 2013). Recently, the IFNL4 gene was discovered. It is controlled by a dinucleotide polymorphism, denoted IFNL4 ΔG > TT (rs368234815; c.65_66delAAinsC; p. Glu22AlafsTer25) and located in exon 1 of IFNL4. The IFNL4 ΔG allele creates a novel gene which encodes the interferon-λ4 protein, moderately similar to interferon-λ3. The alternative allele IFNL4 TT does not create this protein. IFNL4 ΔG > TT was identified in high linkage disequilibrium with rs12979860, now more properly called IFNL4 rs12979860 because of its location within intron 1 of IFNL4 (Prokunina-Olsson *et al.*, 2013; O'Brien *et al.*, 2014). IFNL4 ΔG > TT seems to be a better predictor of HCV clearance than IFNL4 rs12979860, mainly in individuals of African ancestry (Prokunina-Olsson *et al.*, 2013; Bibert *et al.*, 2013; O'Brien *et al.*, 2014). The present study aimed to determine the prevalence of the IFNL4 ΔG > TT variants and to evaluate the association with spontaneous clearance of HCV infection in Brazilian HIV-1 positive patients.

The study was performed with HIV-1 positive patients from a cross-sectional study conducted previously (July 2008 to January 2009) in a reference outpatient treatment center for HIV testing and AIDS treatment in Canoas (Rio Grande do Sul, Brazil). A total of 580 adult patients (aged ≥ 18 years) were consecutively enrolled, and 57 refused to participate in the study. Socio-demographic and clinical variables of the studied sample were described previously (Lunge *et al.*, 2012; Simon *et al.*, 2014). All study participants signed an informed consent form. The study was approved by the Research Ethics Committee of the Universidade Luterana do Brasil (process 139H/2007).

Blood samples were collected by venipuncture in 5 mL tubes, using ethylenediamitetraacetic acid (EDTA) as anticoagulant, and centrifuged for plasma and cell separation. All plasma samples were submitted to anti-HCV and HCV-RNA detection, as described previously (Lunge *et al.*, 2012). The viral genotype was identified in all HCV-RNA positive samples. Spontaneous HCV clearance was defined as HCV seropositivity and negative HCV RNA results (< 50 UI/ml) without any hepatitis C specific treatment (either interferon or interferon and ribavirin).

IFNL4 genotyping was performed in all patients with a positive result for anti-HCV. Total DNA of each clinical sample was purified by a standard silica-based procedure (Boom *et al.*, 1990). IFNL4 genotypes were determined by polymerase chain reaction (PCR) using primers (5'-GCCTGCTGCAGAAGCAGAGAT-3' and R 5'-GCTCCAGCGAGCGGTAGTG-3') described previously (Prokunina-Olsson *et al.*, 2013). The amplification mixture consisted of ultrapure distilled water (DNase and RNase free), 10 mM Tris-HCl, pH 8.5, 50 mM KCl, 0.75 mM MgCl_2_, 0.0625 mM of deoxynucleoside triphosphates, 0.25 μM of each primer, 1 U of *Taq* DNA polymerase (Cenbiot Enzimas, Brazil) and 20-100 ng of DNA. All reactions were performed with the following cycling parameters: 1 cycle at 94 °C for 3 min followed by 35 cycles at 94 °C for 10 s, 61 °C for 30 s and 72 °C for 30 s, followed by a final extension step at 72 °C for 5 min. The amplified DNA was digested for restriction fragment length polymorphism (RFLP) analysis using *Ape*K I restriction enzyme according the manufacturer instructions (New England Biolabs). Digested fragments were separated by electrophoresis on a 10% polyacrylamide gel stained with silver nitrate. The IFNL4 TT allele presented three fragments (of 92, 32 and 5 bp, respectively), while the IFNL4 ΔG allele presented four fragments (of 75, 32, 16 and 5 bp, respectively). PCR amplified products of the three IFNL4 genotypes were sequenced to validate the PCR-RFLP assay results.

Data were expressed as mean and standard deviation (± S.D.) or frequency percentage (%). Variables were compared between groups using Student's *t*-test or the nonparametric Mann-Whitney test for categorical variables, and the chi-square test for qualitative variables. Allele frequencies were obtained by direct counting of alleles. Allele and genotype frequencies were compared between groups using the chi-square test. The linkage disequilibrium between IFNL4 ΔG > TT and IFNL4 rs12979860 polymorphisms was calculated using the CubeX program (Gaunt *et al.*, 2007). All tests were two-tailed and *P* < 0.05 values were considered statistically significant.

Out of 580 HIV-1 positive patients originally included in this study, 138 (23.8%) patients had an HCV-positive result. Sociodemographic traits, risk factor for HIV-1, and clinical characteristics of HIV-1/HCV co-infected patients are presented in Table 1. The mean age of this group of HCV-positive patients was 41.7 ± 9.5 years, and 62.3% were men. Spontaneous clearance of the HCV infection was observed in 34 individuals (24.6%). HCV genotype analysis in the chronically infected individuals showed that 63 (60.6%) patients had genotype 1, 5 (4.8%) had 2 genotype, and 36 (34.6%) had genotype 3. The mean log_10_ HCV-RNA was 6.8 ± 0.7, 6.9 ± 0.3, and 6.6 ± 0.9 IU/ml for HCV genotypes 1, 2, and 3, respectively (p=0.14). The IFNL4 genotypes were not associated with HCV viral load.

**Table 1:** Socio-demographic and clinical characteristics of the HIV/HCV co-infected study population.

Variable	Total^a^	Chronic infection	Spontaneous clearance	p
	(n = 138)	(n = 104)	(n = 34)	
Age (years)	41.7 ± 9.5	41.4 ± 9.5	42.7 ± 9.5	0.49
Male gender	86 (62.3)	64 (62.5)	21 (61.8)	> 0.99
White skin color	82 (59.4)	65 (62.5)	17 (50.0)	0.28
CD4+ count (cells/mm^3^)	429 ± 243	415 ± 225	472 ± 295	0.30
HIV viral load (log_10_ copies/ml)	2.7 ± 1.3	2.6 ± 1.2	2.8 ± 1.4	0.42
Time since HIV diagnosis (years)	6.2 ± 3.7	6.1 ± 3.7	6.5 ± 3.7	0.59
HAART use^b^	101 (82.1)	76 (82.6)	25 (80.6)	0.81
Injecting drug use	55 (39.9)	37 (35.6)	18 (52.9)	0.07
HCV viral load (log_10_ copies/ml)		6.8 ± 0.7	NA	
HCV genotype			NA	
*1*		63 (60.6)		
*2*		5 (4.8)		
*3*		36 (34.6)		

HIV: human immunodeficiency virus; HCV: hepatitis C virus; HAART: highly active antiretroviral therapy; NA: not applicable

a Data are reported as mean and standard deviation (± S.D.) or frequency and percentage (%).

b Totals do not coincide due to lack of data from participants in the study (n=15).

Allele and genotype frequencies of the IFNL4 ΔG > TT polymorphism in the HIV-1/HCV co-infected patients are presented in Table 2. The IFNL4 ΔG allele frequency was 37%. The genotypic frequencies are in Hardy-Weinberg equilibrium for the total sample. A significant difference was observed between HCV chronic infected patients and individuals with spontaneous clearance (p=0.002). The probability of spontaneous resolution of the HCV infection in patients with IFNL4 TT/TT genotype was 3.6 higher than for patients carrying ΔG allele (OR = 3.63, 95% CI: 1.51-8.89; p=0.001). The distribution of IFNL4 ΔG > TT genotypes in comparison to IFNL4 rs12979860 genotypes is shown in Table 3. The variants are in strong linkage disequilibrium (D' = 0.883; r^2^ = 0.733).

**Table 2:** Allele and genotype frequencies of the rs368234815 *IFNL4* gene polymorphism in the study population.

	Total^a^	Chronic infection	Spontaneous clearance	*P* value^b^
	(n = 138)	(n = 104)	(n = 34)	
Allele model				0.039
ΔG	102 (37.0)	84 (40.4)	18 (26.5)	
TT	174 (63.0)	124 (59.6)	50 (73.5)	
Genotype model				0.002
ΔG/ΔG	17 (12.3)	12 (11.5)	5 (14.7)	
TT/ΔG	68 (49.3)	60 (57.7)	8 (23.5)	
TT/TT	53 (38.4)	32 (30.8)	21 (61.8)	
ΔG dominant model^c^				0.001
ΔG/ΔG + TT/ΔG	85 (71.6)	72 (69.2)	13 (38.2)	
TT/TT	53 (38.4)	32 (30.8)	21 (61.8)	

a Data are number (%) of patients.

b Chi-square test; *P* values refer to comparison between chronic infection and spontaneous clearance.

c ΔG dominant model: OR = 3.63, 95% CI: 1.51-8.89.

**Table 3:** The *IFNL4* rs368234815 and rs12979860 genotypes in the study population.

*IFNL4* rs368234815	*IFNL4* rs12979860		
	CC	CT	TT
ΔG/ΔG	1	-	16
TT/ΔG	5	63	-
TT/TT	42	11	-

Linkage disequilibrium results between *IFNL4* variants: D' = 0.883; r2 = 0.733.

Previous studies demonstrated that the IFNL4 rs12979860 polymorphism was significantly associated with spontaneous clearance and response to therapy in HCV infected patients (Ge *et al.*, 2009; McCarthy *et al.*, 2010). However, recent studies reported that the IFNL4 ΔG > TT polymorphism has a better prediction value than IFNL4 rs12979860. The IFNL4 ΔG > TT polymorphism has been proposed as a candidate to provide a causal link between variants nearby IFNL3 or within IFNL4 and spontaneous clearance of the HCV, thus solving some confusing results (Covolo *et al.*, 2014). In the present study we observed that the genotype distribution was significantly different between HCV chronic infection patients and individuals who had spontaneous clearance. There was an association between the IFNL4 TT/TT genotype of the IFNL4 ΔG > TT polymorphism and spontaneous clearance. In our previous study, the HIV-1/ HCV co-infected group presenting the CC genotype of the IFNL4 rs12979860 polymorphism had a 2.8 times higher probability for spontaneous clearance than the other genotypes (Lunge *et al.*, 2012). The current results now showed that patients with the IFNL4 TT/TT genotype had a 3.6 times higher probability for spontaneous HCV clearance than patients carrying the ΔG allele. Our finding is in agreement with other studies that investigated the association of the IFNL4 ΔG > TT polymorphism with impaired spontaneous or treatment-induced clearance of HCV in HIV-1 infected patients. A cohort of 207 patients treated with interferon was investigated and the IFNL4 ΔG > TT polymorphism was a better predictor of treatment failure than IFNL4 rs12979860 (Franco *et al.*, 2014). Similar results were found in an analysis of 890 HIV-1/HCV co-infected women. HCV clearance was three-fold higher in black women with the IFNL4 TT/TT genotype than in those with TT/ΔG or ΔG/ΔG genotypes (Aka *et al.*, 2014). However, another study that analyzed 206 HIV/HCV co-infected patients and 162 HCV mono-infected patients found that the IFNL4 ΔG > TT polymorphism was strongly associated with the response to interferon/ribavirin therapy in mono-infected patients, but not in co-infected ones (Krämer *et al.*, 2013).

Other groups have studied this polymorphism in HCV mono-infected patients only. In a Swiss cohort of 540 HCV patients, the IFNL4 ΔG > TT polymorphism was a better predictor of HCV clearance than IFNL4 rs12979860 (Bibert *et al.*, 2013). Interestingly, these authors attributed the effect of the IFNL4 ΔG variant to reduced expression of IFNL3 and interferon-γ-inducible protein-10 (IP-10). In Italian patients, the IFNL4 ΔG > TT polymorphism was significantly associated with a marker for ISG activation (IP-10), but with regard to spontaneous and treatment-induced clearance, IFNL4 ΔG > TT had a predictive value similar to IFNL4 rs12979860 (Covolo *et al.*, 2014). Two other studies also reported similar predictive values of IFNL4 ΔG > TT and IFNL4 rs12979860 polymorphisms (Keshvari *et al.*, 2014; Stättermayer *et al.*, 2014). Nonetheless if, as reported, one accepts the argument that IFNL4 is a functional polymorphism in the process of HCV clearance, then it would make sense to include IFNL4 ΔG > TT genotyping in clinical decisions (Keshvari *et al.*, 2014).

Studies reported that IFNL4 ΔG > TT and IFNL4 rs12979860 polymorphisms are in linkage disequilibrium, but this association varies according to ethnic group. The IFNL4 ΔG allele is completely correlated with the unfavorable IFNL4 rs12979860 T allele in Asians (r^2^ = 1.00) and highly so in Europeans (r^2^ = 0.92). However, in Africans, this correlation is only moderate (r^2^ = 0.71). The IFNL4 ΔG > TT polymorphism has been highly associated with HCV clearance in individuals of African ancestry, with the IFNL4 ΔG allele being better than the IFNL4 rs12979860 T allele in predicting impaired clearance and treatment failure in HCV infection (Prokunina-Olsson *et al.*, 2013). The allele frequencies of both polymorphisms also differ according to ethnic groups. According to the HapMap project (Prokunina-Olsson *et al.*, 2013; Franco *et al.*, 2014), the frequencies for the IFNL4 ΔG allele are 7% in Asians, 32% in Europeans, and 78% in Africans. The Brazilian population is remarkably heterogeneous, which reflect its history of extensive admixture, mainly from Europeans, Africans and Amerindians (Cavalcante *et al.*, 2011). In this regard, the present study comprised a sample of individuals of mixed ancestry, but the majority (59.4%) of the subjects self-reported skin color as white. The frequency of IFNL4 ΔG > TT polymorphism in the Brazilian populations is not known yet. Our study found a frequency of 37% for the IFNL4 ΔG allele, which is associated with impaired HCV clearance. The frequency of the IFNL4 ΔG allele in our study was similar to that reported in Swiss (38%) (Bibert *et al.*, 2013) and European (32%) (Franco *et al.*, 2014) cohorts.

Certain limitations must be considered in this study. First, the statistical power to detect differences was limited due to small sample size, which prevented us to perform a multivariate analysis. Second, it was not possible to identify the time of infection for the two viruses (HCV and HIV-1) and to clarify the precise relation between HIV-1 infection and HCV clearance.

To investigate factors that influence spontaneous clearance of HCV, as well as clearance induced by IFN-based treatment is important to understand the natural history of infection and establish new insights about treatment strategies. It has been reported that IFNL4 polymorphisms are associated with treatment outcomes based on direct-acting antivirals (DAAs) (Chu *et al.*, 2012; Zeuzem *et al.*, 2013; Meissner *et al.*, 2014). However, IFN-free DAA treatments allow high cure rates, but come with matching high price tags (The Lancet, 2014). In this sense, IFNL4 genotyping could to be useful tool before and until global access to DAA can be achieved, helping to prioritize DAA treatment.

In conclusion, our findings suggest that the IFNL4 ΔG > TT polymorphism of the IFNL4 gene seems to be better than IFNL4 rs12979860 to predict spontaneous clearance of the HCV in HIV-1 positive patients.
